# Definition of Mucosal Breaks in the Era of Magnifying Endoscopy with Narrow-Band Imaging

**DOI:** 10.1155/2022/3952962

**Published:** 2022-05-13

**Authors:** Daisuke Kikuchi, Hiroyuki Odagiri, Yoshio Hoshihara, Yorinari Ochiai, Yugo Suzuki, Junnosuke Hayasaka, Masami Tanaka, Kosuke Nomura, Satoshi Yamashita, Akira Matsui, Toshiro Iizuka, Shu Hoteya

**Affiliations:** Department of Gastroenterology, Toranomon Hospital, Japan

## Abstract

**Background:**

Gastroesophageal reflux disease is diagnosed endoscopically based on the presence of mucosal breaks. However, mucosal breaks can be judged differently depending on the endoscopist, even in the same image. We investigated how narrow-band imaging (NBI) and magnified endoscopy affect the judgment of mucosal breaks.

**Methods:**

A total of 43 consecutive patients were enrolled who had suspected mucosal breaks on white-light images (WLI) and underwent nonmagnified NBI (N-NBI) and magnified NBI (M-NBI) by a single endoscopist. From WLI, N-NBI, and M-NBI, 129 image files were created. Eight endoscopists reviewed the image files and judged the presence of mucosal breaks.

**Results:**

The 8 endoscopists determined mucosal breaks were present in 79.4 ± 9.5% (67.4%–93.0%) on WLI, and 76.7 ± 12.7% (53.5%–90.7%) on N-NBI. However, the percentage of mucosal breaks on M-NBI was significantly lower at 48.8 ± 17.0% (18.6%–65.1%) (*p* < 0.05). Intraclass correlation between observers was 0.864 (95% CI 0.793–0.918) for WLI and 0.863 (95% CI 0.791–0.917) for N-NBI but was lower for M-NBI at 0.758 (95% CI 0.631–0.854).

**Conclusion:**

Rates of detection and agreement for mucosal breaks on WLI and N-NBI were high among endoscopists. However, these rates were lower on M-NBI.

## 1. Background

Gastroesophageal reflux disease (GERD) has recently become increasingly common in Japan due to factors such as the decreased rate of *Helicobacter pylori* infection and westernization of the Japanese diet, and it is an extremely common condition encountered in routine practice [1–3]. GERD is defined as a disease in which gastroesophageal reflux causes esophageal mucosal injury and/or troublesome symptoms. In other words, both endoscopic findings and symptoms are important in the diagnosis of GERD. GERD is classified as erosive if esophageal mucosal injury is observed endoscopically and as nonerosive if only symptoms are present. The Los Angeles (LA) classification is widely used for classifying the severity of erosive GERD based on endoscopic findings [4]. The LA classification proposes the concept of mucosal breaks, which are different from conventionally referenced endoscopic findings such as erosions and ulcers.

The image resolution of gastrointestinal endoscopy has increased dramatically in recent years [5, 6]. In addition, advances in image-enhanced and magnifying endoscopy techniques such as narrow-band imaging (NBI) and blue laser imaging (BLI) have made detailed observation feasible for the first time. The endoscopic images that can be obtained with the latest technology are completely different from those that could be obtained when the LA classification was first proposed. A mucosal break is defined as “an area of slough or erythema with a discrete line of demarcation from the adjacent more normal looking mucosa” but sometimes the presence of mucosal breaks is judged differently among endoscopists. In this study, we investigated differences in endoscopists' judgments of mucosal breaks on white-light images (WLI), nonmagnified NBI, and magnified NBI.

## 2. Methods

### 2.1. Patients

This study involved 43 consecutive patients with mucosal breaks suspected on WLI, who then underwent nonmagnified NBI and magnified NBI for close examination of the suspected breaks between April 2016 and April 2018. The same endoscopist (D.K.) performed all examinations by WLI, nonmagnified NBI, and magnified NBI. Based on a comprehensive assessment combining nonmagnified NBI and magnified NBI, mucosal breaks suspected on WLI were confirmed in 28 of 43 patients but were judged to be absent in the remaining 15 patients.

This study was conducted with the approval of the Institutional Review Board of Toranomon Hospital and a research grant from the Okinaka Memorial Institute for Medical Research.

### 2.2. Endoscopic Examinations

The endoscopes used in this study were the GIF-H260Z and GIF-H290Z (Olympus Corporation). Sedatives were generally not used during endoscopy, but appropriate doses of pethidine hydrochloride or diazepam were used as needed. Scopolamine butylbromide was used as an antispasmodic.

Patients were instructed to take deep breaths as the lower esophagus was insufflated to fully dilate the esophagogastric junction (EGJ) for imaging. After the patients took at least 3 deep breaths, the EGJ was first imaged by WLI. When a clearly demarcated area of erythema suspected to be a mucosal break was observed, the area was then imaged by nonmagnified NBI. Finally, the same area was imaged by magnified NBI.

### 2.3. Image Files

**Figure 1:**
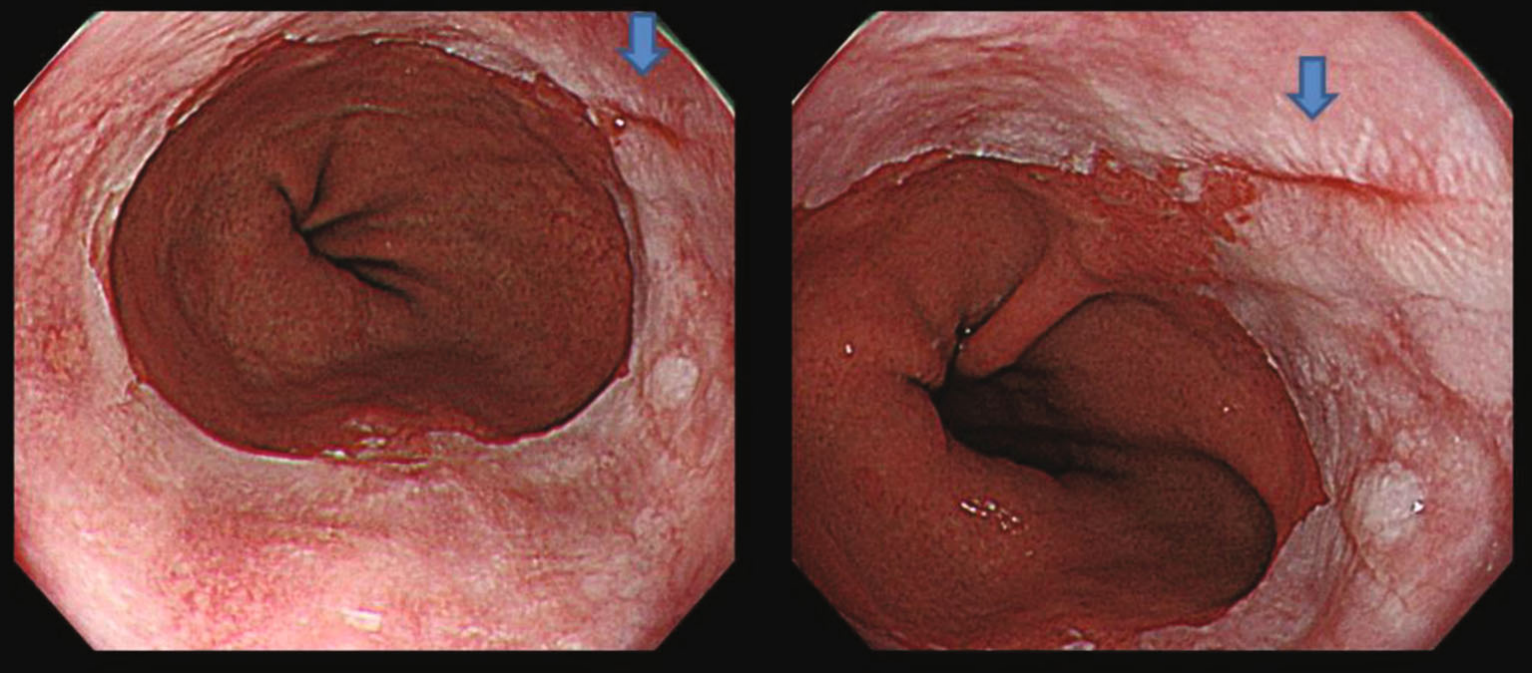
Example image file of white-light endoscopy images used in the study. All 8 endoscopists judged the areas marked with a blue arrow to be a mucosal break.

**Figure 2:**
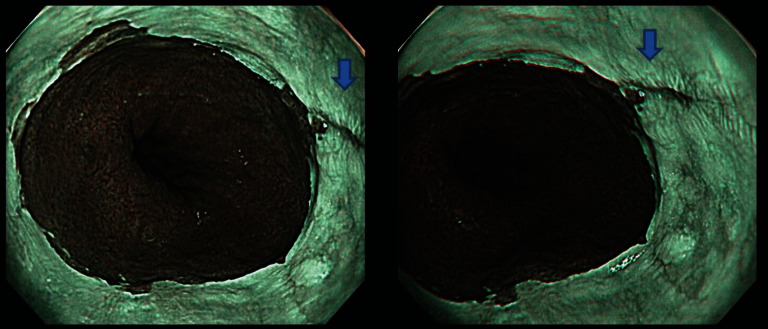
Example image file of nonmagnified narrow-band imaging used in this study (same patient as Figure 1). All 8 endoscopists judged the area marked with a blue arrow to be a mucosal break.

**Figure 3:**
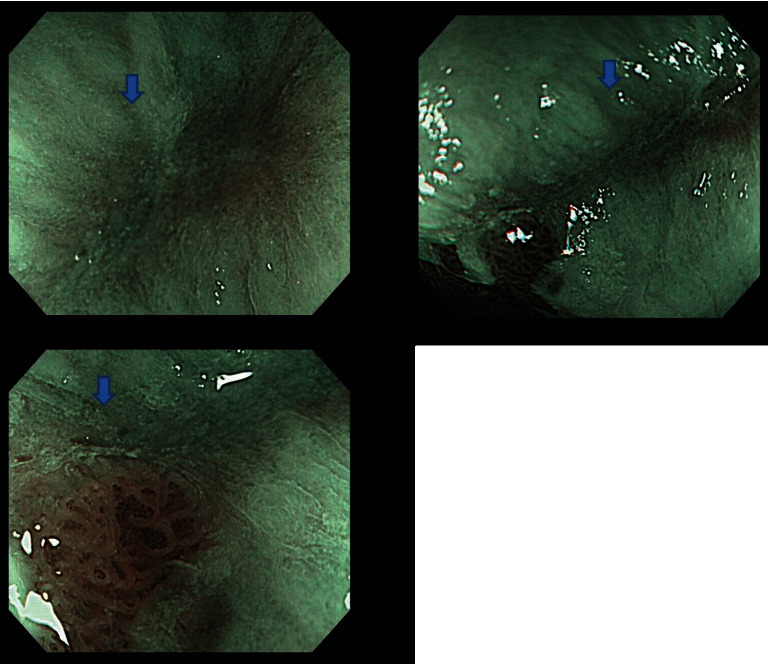
Example image file of magnified narrow-band imaging used in this study (same patient as Figures 1 and 2). The areas of sharply demarcated erythema noted on white-light images were completely epithelialized (blue arrows), and all 8 endoscopists judged the images not to show a mucosal break.

Image files of the EGJ of the 43 patients were created by a single endoscopist (D.K.). Three image files were created for each patient: 1 for WLI (Figure 1), 1 for nonmagnified NBI (Figure 2), and 1 for magnified NBI (Figure 3). A total of 129 files were created. Images captured with almost the same field of view were selected as images for WLI and nonmagnified NBI, and images moderately to highly magnified at the site of a mucosal break were selected as images for magnified NBI. The area of the suspected mucosal break to be assessed by each endoscopist was marked with a blue arrow. Each image file contained 2–4 endoscopic images.

### 2.4. Image Evaluation

Eight endoscopists evaluated the image files presented in random order. Four of the 8 endoscopists were board certified by the Japan Gastroenterological Endoscopy Society, and 4 were not. Before evaluation, they were blinded to the patients' clinical information and the judgments of the other endoscopists. Endoscopists determined the presence of mucosal breaks on WLI when they found “an area of slough or erythema with a discrete line of demarcation from the adjacent more normal looking mucosa,” as in the LA classification. In addition, the endoscopists were instructed to judge the presence of mucosal breaks on nonmagnified NBI and magnified NBI as they would for similar findings in routine clinical practice.

The primary endpoint was the percentage of mucosal breaks on each imaging modality. The secondary endpoint was the intraclass correlation for the rate of agreement between endoscopists. Also, the intraclass correlation of the rate of agreement for diagnosis of mucosal breaks by each modality was examined for board-certified endoscopists and nonboard-certified endoscopists.

### 2.5. Statistics

Data were analyzed using the unpaired *t*-test and chi-squared test as appropriate. A *p* value less than 0.05 was considered significant. Intraclass correlation was calculated and all statistical analyses were performed using SPSS version 20 (SPSS IBM statistics).

## 3. Results

The mean age of the 43 patients was 65 ± 22 years. Thirty-one were male and 12 were female. Four patients were given pethidine, and 2 were given diazepam. Twenty-five patients were using a proton pump inhibitor before endoscopy (Table 1).

**Figure 4:**
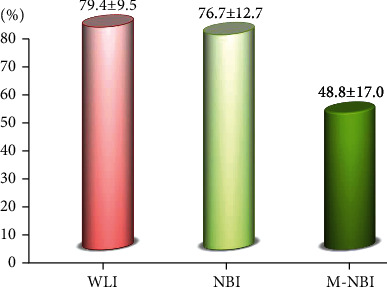
Percentages of mucosal breaks on WLI, nonmagnified NBI, and magnified NBI. NBI: narrow-band imaging; WLI: white-light imaging.

The endoscopist who performed the endoscopies recorded that a mucosal break was present in the final assessment on medical records in 65.1% (28/43) of the patients. The percentage of image files that the 8 reviewing endoscopists determined to have a mucosal break did not differ significantly between WLI (79.4 ± 9.5% [67.4%–93.0%]) and nonmagnified NBI (76.7 ± 12.7% [53.5%–90.7%]), but was significantly lower for magnified NBI (48.8 ± 17.0% [18.6%–65.1%]; *p* < 0.05) (Figure 4).

**Figure 5:**
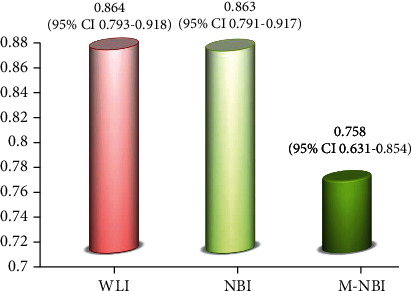
Intraclass correlation between observers. NBI: narrow-band imaging; WLI: white-light imaging.

Intraclass correlation between observers was 0.864 (95% confidence interval [CI] 0.793–0.918) for WLI and a comparably high 0.863 (95% CI 0.791–0.917) for nonmagnified NBI but was lower for magnified NBI at 0.758 (95% CI 0.631–0.854) (Figure 5).

The intraclass correlation of board-certified endoscopists and that of nonboard-certified endoscopists were, respectively, 0.870 (95% CI, 0.732-0.940) and 0.737 (95% CI, 0.495-0.873) for WLI, 0.878 (95% CI, 0.366-0.982) and 0.706 (95% CI, 0-0.953) for nonmagnified NBI, and 0.800 (95% CI, 0.115-0.969) and 0.600 (95% CI, 0-0.932) for magnified NBI.

## 4. Discussion

The LA classification system has long been widely used for endoscopic diagnosis of GERD [1]. GERD had conventionally been diagnosed mainly by the presence of erosion or ulcer until the concept of mucosal breaks was proposed in the LA classification. Since the proposal of LA classification, endoscopic diagnosis of GERD has been based on the presence of mucosal breaks. GERD is also classified as grade A to D according to the length or extent of mucosal breaks. Grade A is defined as “mucosal injury limited to the mucosal folds that is no larger than 5 mm in extent.” If grades A and B are considered mild and grade C and D severe, then most Japanese patients would have a mild form of GERD [7].

One problem with the LA classification is its poor correlation with symptoms. The LA classification was shown to be strongly correlated with heartburn symptom severity when it was first proposed, but later research showed that it is not always highly correlated with symptom severity. Nagahara et al. divided patients with endoscopically proven GERD into groups by symptoms and found that a relatively high percentage (11.6%) overall, and a particularly high percentage of elderly patients, were asymptomatic [7]. In addition, Okamoto et al. found that even 40% of patients with severe (grade C/D) GERD were asymptomatic, even though heartburn symptoms are considered a significant predictive factor for erosive GERD [8]. A new system needs to be developed for grading severity based on endoscopic findings that correlate well with symptom severity.

Another problem with the LA classification is the rate of agreement between endoscopists [9–11]. Many studies have investigated the rate of agreement between endoscopists in grading GERD as grade A or B based on whether the mucosal break is 5 mm or smaller or is larger than 5 mm. It has also been reported that the agreement rate improves for more experienced endoscopists [10]. The rate of agreement for LA classification grades is important but improving the rate of agreement for determining the presence of a mucosal break is even more important. What one endoscopist considers to be a mucosal break, another endoscopist might consider not to be a mucosal break. Research using standardized endoscopy conditions is necessary to address this problem. Detailed observation of the EGJ is difficult in excessively sedated patients because the junction does not dilate. In addition, the technique used by the endoscopist can sometimes result in poor imaging of the EGJ [12]. Thus, in this study, we had other endoscopists evaluate images captured by a single endoscopist. Notably, there is a major discrepancy in resolution between the endoscopic images captured when the LA classification was proposed in the 1990s and the images that can be captured today with image-enhanced endoscopy and magnifying endoscopy techniques such as NBI and BLI. We believe it is very important to understand how different endoscopists judge the same modern-day, high-resolution, endoscopic images.

NBI and BLI have become widely used for endoscopic diagnosis of superficial gastrointestinal neoplasms in recent years. NBI in particular has been shown to be more useful than WLI for detecting esophageal cancer [13]. Studies have reported that using NBI in endoscopic diagnosis of GERD also improves the detection rate of mucosal breaks, as well as the inter-rater agreement rate [14, 15]. However, these studies compared nonmagnified NBI with WLI. This is likely because areas of erythema are more clearly noticeable as brownish areas on NBI than on WLI. However, endoscopists judge NBI images of suspected mucosal breaks on WLI differently depending on whether the images are magnified or not. When endoscopists reviewed WLI and nonmagnified NBI images of suspected mucosal breaks observed by a single endoscopist on WLI, they determined mucosal breaks to be present at about the same rate on each of the two modalities, and the interobserver agreement rate was high. However, fewer endoscopists determined mucosal breaks to be present on magnified NBI, and the interobserver agreement was lower. This is likely because endoscopists judged completely epithelialized areas of erythema differently. The frequency of mucosal breaks may depend on whether they are assessed by nonmagnified endoscopy alone or comprehensively by combination with magnified NBI. Further research will be required to establish a standardized method for the assessment of mucosal breaks. The LA classification provides definitions of mucosal breaks diagnosed by WLI only. Given the widespread use of nonmagnified NBI and magnified NBI in routine practice, comprehensive definitions based on findings of WLI, nonmagnified NBI, and magnified NBI may also be required. Moreover, training in which endoscopists learn the characteristics of lesions for diagnosing mucosal breaks is important. In this study, the intraclass correlations in all modalities were higher for board-certified endoscopists than for nonboard-certified endoscopists. Also, the intraclass correlation was lowest for magnified NBI irrespective of whether endoscopists were board certified. Although this study's sample size was small, our findings indicate that training is extremely important for endoscopists to assess mucosal breaks, especially by magnified NBI.

Our study has a few limitations. The first is that it was a small, retrospective study. Another major limitation is that we used still images only. Endoscopic images of the EGJ change depending on inhalation and the volume of gas used for insufflation. Prospective studies or video-based studies are needed to make judgments in the future. The relationship with symptoms should also be investigated. Studies have shown that the endoscopic diagnosis of GERD is not always strongly correlated with symptoms, and future studies will need to investigate the correlation between diagnostic criteria using image-enhanced endoscopy or magnifying endoscopy and symptoms. The problem examined in this study was only that using the LA classification, which is the definition of mucosal breaks assessed by WLI alone, is inadequate in routine practice today, where magnified NBI is widely used. For diagnosing mucosal breaks, characteristics of lesions observed by all modalities need to be reviewed in the future.

In conclusion, when endoscopists reviewed images of suspected mucosal breaks captured by a single endoscopist, the percentage of images they determined as showing a mucosal break was comparable between WLI and nonmagnified NBI. The rate of agreement between endoscopists was also high for these modalities. However, when the endoscopists reviewed images of the same area on magnified NBI, the percentage of images they determined as showing a mucosal break decreased significantly, and the endoscopists' judgments varied. This indicates that handling of completely epithelialized areas of erythema in diagnostic criteria needs to be standardized in the modern era of magnified NBI.

## Figures and Tables

**Table 1:** Patient characteristics.

*N*	43
Sex (male/female)	31/12
Age (±SD)	64.1 ± 18.5
Mucosal break in medical record (+/-)	28/15
PPI (+/-)	25/18

SD: standard deviation; PPI: proton pomp inhibitor.

## Data Availability

No data available.
